# Identified *IGSF9* association with prognosis and hypoxia in nasopharyngeal carcinoma by bioinformatics analysis

**DOI:** 10.1186/s12935-020-01587-z

**Published:** 2020-10-12

**Authors:** Donglan Huang, Qianqian Liu, Weijun Zhang, Chunyue Huang, Ronghui Zheng, Guofeng Xie, Hongmei Wang, Baochang Jia, Jianjun Shi, Yawei Yuan, Min Deng

**Affiliations:** 1 Department of Radiation Oncology, Institute of Cancer Research, Affiliated Cancer Hospital & Institute of Guangzhou Medical University, Guangzhou, China; 2 Department of Gynecological Oncology, Affiliated Cancer Hospital & Institute of Guangzhou Medical University, Guangzhou, China

**Keywords:** Nasopharyngeal carcinoma, Bioinformatics analysis, *IGSF9*, Prognosis, Hypoxia

## Abstract

**Background:**

Despite improvements in nasopharyngeal carcinoma (NPC) treatment, patients with recurrence and metastasis still have a poor prognosis. Thus, the identification of novel biomarkers is urgently needed to predict outcomes and tailor treatment for NPC.

**Methods:**

Four data sets were downloaded from Gene Expression Omnibus, and one data set GSE68799 of which was applied to filtrate key modules and hub genes by construction of a co-expression network. Other data sets (GSE12452 and GSE53819) were used to verify hub genes. The data set GSE102349 was devoted to identify prognostic hub genes by survival analysis. To explored whether prognostic hub genes are related to hypoxia signatures in NPC, correlation analysis was carried out, and followed by functional verification experiments of those genes in vitro.

**Results:**

By co-expression network analysis, blue module was regarded as a key module in the benign and malignant group, and *IGSF9* of the blue module was identified as a prognostic hub gene. Moreover, *IGSF9* is expected to be a innovative hypoxia-related gene in NPC based on the strong associativity between expression of *IGSF9* and hypoxia scores of three signatures (99-gene, 26-gene and 15-gene). Further functional studies verified that down-regulated expression of *IGSF9* could reduce the proliferation, migration and invasion ability of NPC cells, and hypoxia could induce the expression of *IGSF9*.

**Conclusion:**

*IGSF9* was identified to be relevant to prognosis and involved in hypoxia in NPC. *IGSF9* might serve as one novel prognostic indicator of NPC in the future.

## Background

Nasopharyngeal carcinoma (NPC) can be found most frequently in southern China and southeast Asian countries. According to the global cancer statistics of 2018, it was estimated that there were 129,079 new NPC cases and 72,987 deaths that year [1]. Despite improved treatment for NPC patients, especially concurrent chemoradiation therapy combined with adjuvant chemotherapy, those patients with recurrence and metastasis still have worse overall survival [2]. Tumor-node-metastasis staging system can provide an effective prognostic prediction for NPC patients [3]. However, this factor fails to be accurate enough for evaluating the survival outcomes.

It is well documented that the number of biomarkers might predict the prognosis of NPC, such as the EBV DNA levels, lactate dehydrogenase (LDH), *SOX2, NGX6, FMNL3* and *ANXA2* [4–9]. However, the molecular mechanism of NPC has not been completely established. Therefore, the identification of novel biomarkers that reflect tumors heterogeneity is urgently needed to predict outcomes and tailor treatment for NPC patients.

It is noted that bioinformatics analysis is commonly used to identify candidate biomarkers from high-throughput data. Weighted gene co-expression network analysis (WGCNA) [10], which is mainly applied to filtrate hub genes from large-scale data sets, has been successfully applied in several human tumors [11–14]. WGCNA had been used to analyze the NPC data sets and identify some candidate diagnostic genes [15, 16], which have limited application in the predication of prognosis in NPC patient.

In this study, we performed WGCNA to identify hub genes and validate these hub genes using three data sets (GSE68799, GSE12452 and GSE53819). Another one data set GSE102349 that contains follow-up information was used for univariate Cox regression analysis to screen hub genes with prognostic values. Numerous reports have testified that tumor hypoxia leads to poor prognosis [17–19]. We hypothesized that one prognostic hub gene (*IGSF9*) might be involved in hypoxia in NPC. Hence, Pearson’s correlation analysis was conducted and the result showed that expression of *IGSF9* was related to the scores of three hypoxia signatures. Furthermore, functional studies confirmed that the elevated expression of *IGSF9* induced by hypoxia increased NPC cells proliferation, invasion and migration in vitro.

## Materials and methods

### Data download and pre-processing

Four NPC micro-array data sets (Additional file 1: Table S1) were downloaded from the well-known gene expression omnibus (GEO) database (https://www.ncbi.nlm.nih.gov/geo/). Since the numerical distribution of raw data value of four data sets is too wide to be used for further analysis, we formulated each raw data value in log_2_ (X + 1), where X is the raw data value, followed by quantiles of them. After normalization, expression values of each gene of data set GSE68799 were ranked from large to small by variance analysis report (VAR) value. The expression profile of data set GSE68799 which contains the top 6000 genes was used for WGCNA.

### Co-expression network construction and identification of key modules and hub genes

The processed matrix of the data set GSE68799 was exploited to construct an unsigned gene co-expression network by WGCNA. Genes were clustered by using the hierarchical clustering method according to the topological overlap measure (TOM). In accordance with the criterion of hybrid dynamic tree, each gene module should contain no less than 50 genes. In line with the dissimilarity of module eigengenes (MEs), the height 0.25 was set as a cut off line to combine some modules. In the process of generating the network, 1000 genes were randomly selected every time. Then, modules with the strongest correlation between MEs in each module and clinical traits were identified as key modules. In a similar way, genes with higher module membership (MM) and higher gene significance (GS) were considered as hub genes, filtered criteria was set as MM > 0.8 and GS >|0.7| [14].

### Validation of hub genes

Differentially expressed genes (DEGs) between NPC group and non-NPC group of three data sets (GSE68799, GSE12452 and GSE53819) were screened by the “limma” R package. Cut-off value was determined by adjusted *P* < 0.05. Validated hub genes were obtained by overlapping hub genes and common DEGs. Comparison analysis for expression of validated hub genes was conducted in benign and malignant groups of three data sets (GSE68799, GSE12452 and GSE53819), and the same analysis for expression of validated hub genes was performed in different clinical stages of the data set GSE102349.

### Identification and functional analysis of prognostic hub genes

The profile contains 23,706 genes from the data set GSE102349 was exploited for univariate Cox regression analysis by the “survival” R package. Cut-off value was set as hazard ratio (HR) >|1|, and adjusted P < 0.05. If one validated hub gene was significantly associated with progression-free survival (PFS), it could be identified as a prognostic hub gene. Functional analysis of them was proceeded with gene set enrichment analysis (GSEA) (https://www.gsea-msigdb.org/gsea/index.jsp) [20]. This analysis was performed on the version of MSigDB.v7.0, and each analysis would conduct on 1000 times gene set substitution. Significant pathways were identified by follow criterion: absolute value of normalized enrichment score (NES) > 1, P < 0.05.

### Correlation of hypoxia signatures and prognostic hub genes

To explored whether prognostic hub genes are related to hypoxia signatures in NPC, we focused on three previously reported hypoxia signatures (99-gene, 26-gene and 15-gene) [21–23], which have been regarded as effective prognostic tools in head and neck cancer. The data set GSE102349 was used to summarize the scores of hypoxia signatures. For each gene of hypoxia signatures, each sample in the data set was given a primary score of either + 1 or − 1 as the following criteria [24]: If the expression of that gene of one sample is greater than the median value of that gene of all samples in the data set, this sample would be marked as + 1 score. If not, the sample would be marked as − 1 score. For each sample, the summed value was calculated from the + 1′s and − 1′s of total genes of hypoxia signatures, which was the scores of hypoxia signatures for that sample in data set GSE102349. After that, the scores of hypoxia signatures were exploited for survival analysis and correlation analysis between the scores and the expression of prognostic hub genes and parallel analysis was performed in *P4HA1* which was one of the common genes from three signatures.

### Cell culture and siRNA transfection

CNE-2 and 5-8F cells were cultured in RPMI-1640 medium supplemented with 10% fetal bovine serum (FBS, Gibco BRL, NY, USA) and incubated in a humidified atmosphere at 37 °C with 5% CO_2_. For hypoxia exposure, they were incubated in a anoxic chamber at 37 °C with 5% CO_2_ and 1% O_2_ for 24 h. Two siRNA oligonucleotides (siIGSF9#1 siIGSF9#2) and negative control were purchased from Sangon Biotech (Shanghai, China) and LipofectamineTM 2000 transfection reagent were from Invitrogen (Camarillo, USA).

### Quantitative real time polymerase chain reaction (qRT-PCR)

Total RNA was extracted from cells using TRIzol reagent (Invitrogen) based on the product specification. qRT-PCR was executed using SYBR green qPCR Master Mix (Takara Bio, Otsu, Japan). Comparative CT method was used for quantitative analysis and *GAPDH* was served as the internal reference. Primer sequences as follow: *IGSF9* sense 5′-TCTGTGGACGAGAACTATGAGTG-3′, antisense 5′-AGCCGAGCCCTAGTAGCAT-3′; *P4HA1* sense 5′-GGGACAAGCCTCGTATT-3′, antisense 5′-TTGGGTTTGAAATGGTG-3′; *GAPDH* sense 5′-GACTCATGACCACAGTCCATGC-3′, antisense 5′-AGAGGCAGGGATGATGTTCTG-3′.

### Western blotting

Proteins from cells were lysed using RIPA lysate (Beyotime, Shanghai, China), and quantified using Pierce BCA Protein Assay Kit (Thermo Fisher Scientific, MA, USA). Protein lysates were separated by 10% sodium dodecyl sulfate containing polyacrylamide gels and transferred onto a polyvinylidene difluoride membrane (Millipore, MA, USA) by electrophoresis. GAPDH antibody (1:5000, Abcam, MA, USA), IGSF9 antibody (1:1000, Ybscience, Shanghai, China) and P4HA1 antibody (1:1000, Invitrogen) were incubated at 4℃ overnight. Horseradish Both peroxidase-labeled anti-rabbit (1:8000, Santa Cruz, CA, USA) and anti-mouse (1:8000, Santa Cruz) were used at 4 °C for 2 h. The bands on the membrane were visualized by enhanced chemiluminescence (Thermo Fisher Scientific).

### Cell proliferation, migration and invasion assays

Cell proliferation was estimate using cell counting kit-8 (CCK8) assay. 1000 cells were seeded in per well of 96-well plates. The 450 nm absorbance of each well was detected using CCK8 reagent (Dojindo, Kumamoto, Japan) incubation for 0, 24, 48, 72 and 96 h. Cell migration was evaluated using wound-healing assays. An artificial scratch was conducted on fused monolayer of adherent cells. 48 h later, photographs were got from an inverted microscope (Olympus,Tokyo, Japan). For cells invasion assay, cells were seeded on the matrigel (Corning, NY, USA) of the upper layer of transwell chamber without serum medium. Then, FBS was added into the lower layer of the transwell chamber. After 48 h, non-invasive cells and matrigel were softly removed, while invasive cells that pass through matrigel into the lower layer were colored with crystal violet, counted and imaged.

### Statistical analysis

Data were expressed as mean ± standard deviation (SD) and the difference between two groups was estimated using two-tailed Students’ t-test. Survival difference was evaluated using Log-rank test. Pearson’s correlation analysis was used to calculate the correlation between two continuous variables. *P* < 0.05 was defined to be statistically significant.

## Results

### Identification of two key modules and 79 hub genes

**Fig. 1:**
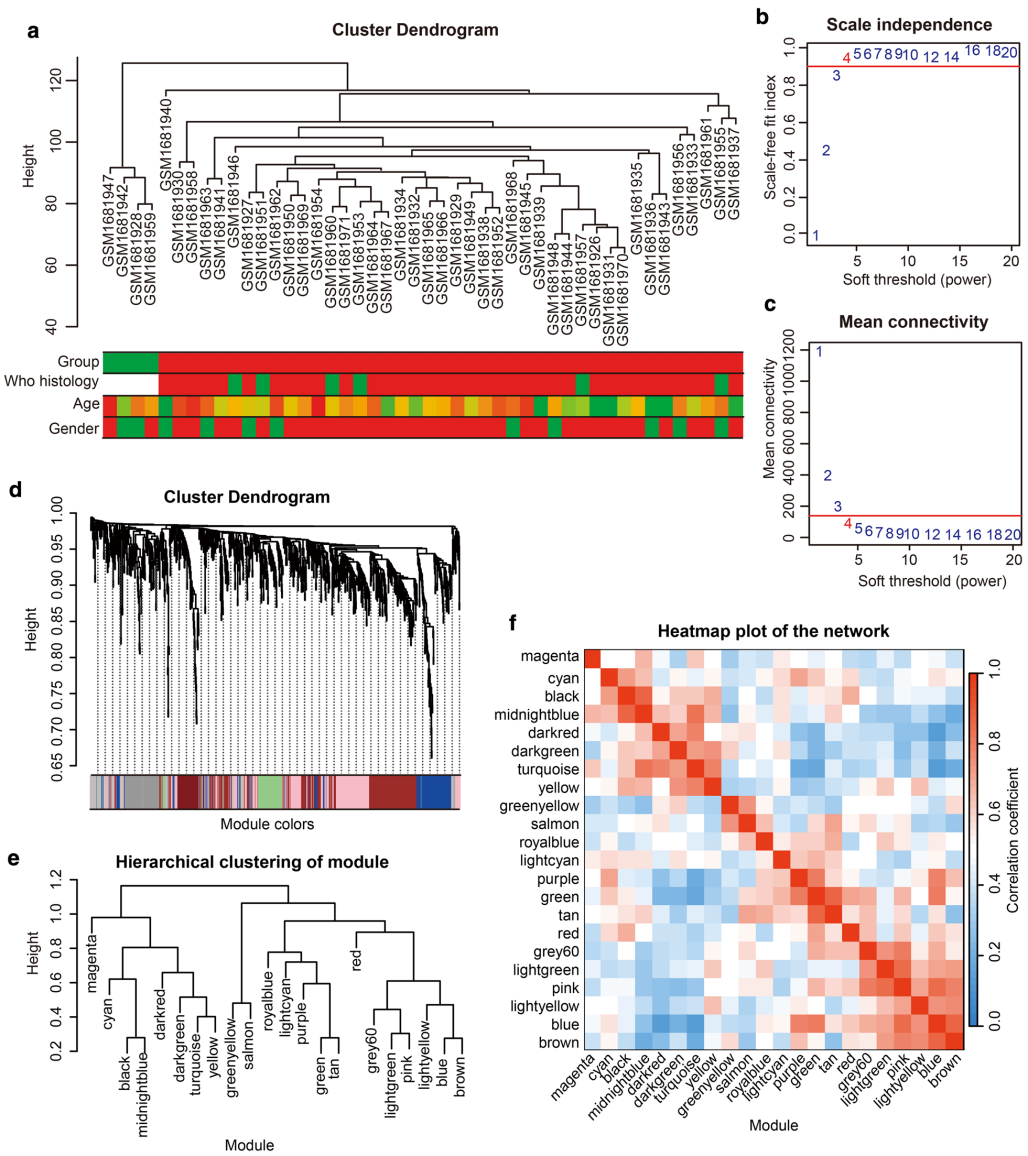
Construction of a co-expression network. **a** Hierarchical clustering was used in the data set GSE68799, which included 42 NPC samples and four non-NPC samples. Matrix of the top 6,000 genes from the data set GSE68799 in order of their VAR values was used for WGCNA. Different colors represent different kinds of clinical traits (Group of benign and malignant, WHO histology, Age and Gender). **b**, **c** Scale-free fit index (**b**) and mean connectivity (**c**) for various soft-thresholding powers. **d** Dendrogram clustered according to a dissimilarity measure (1-TOM). Different branches represent different genes, and different colors below represent different modules. **e** Plot of hierarchical clustering of the module. **f** Heat-map plotted according to adjacencies of the network

**Fig. 2:**
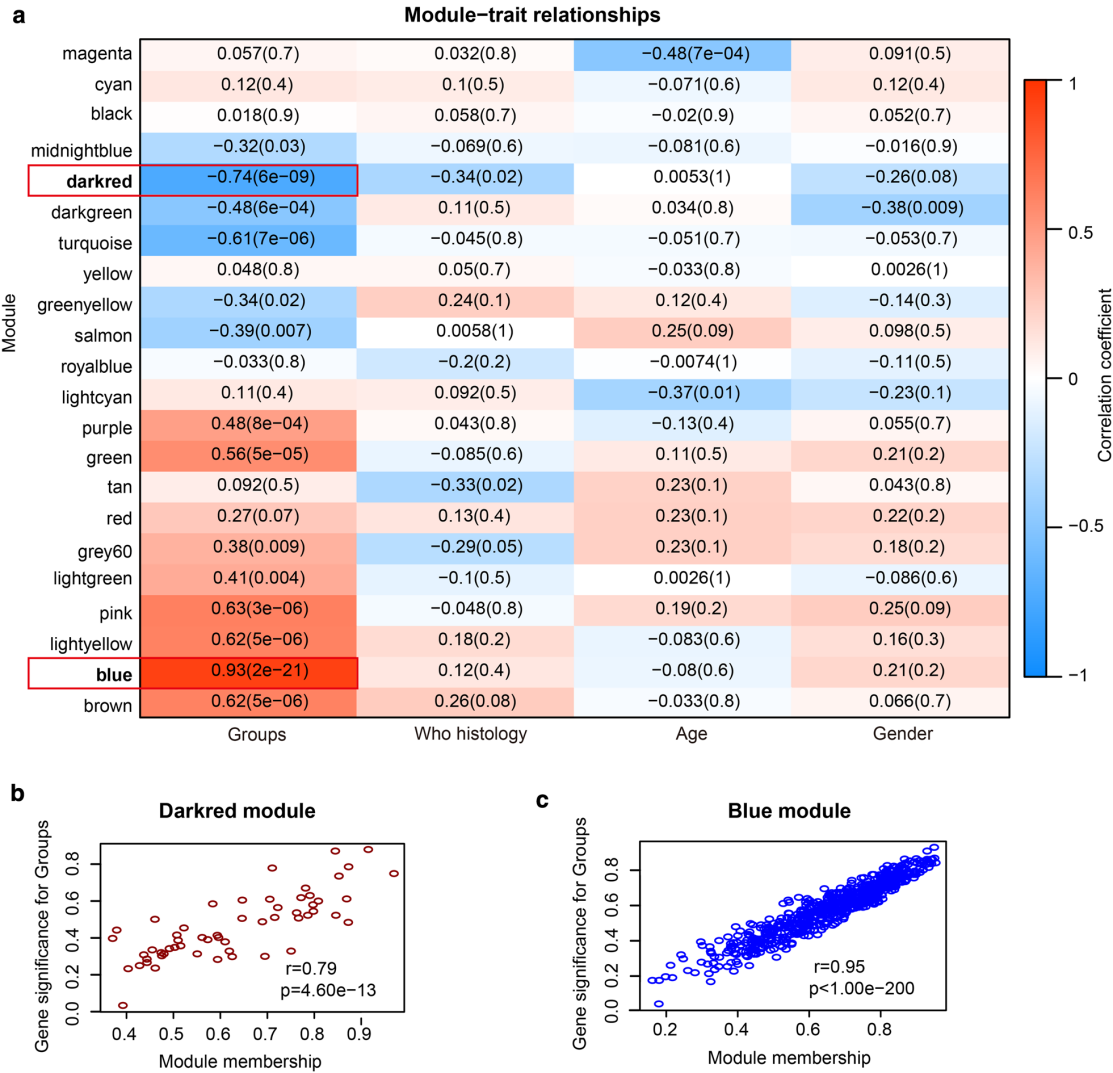
Identification of two key modules and 79 hub genes. **a** Heat-map of the correlation between modules and clinical traits (Group of benign and malignant, WHO histology, Age and Gender) of NPC. Each cell corresponds the correlation. Color legend on the right of the heat-map. Modules framed in red (blue module and dark red module) were considered as key modules. **b, c** Scatter plots of MEs in the dark red module (**b**) and the blue module (**c**)

Expression value of all genes of four NPC data sets were normalized by log_2_ (X + 1) and quantiles (Additional file 1: Fig. S1). The data profile of the top 6,000 genes collated by the VAR values from the data set GSE68799 was used for WGCNA, and 46 samples of this data set could be divided into two clusters (Fig. 1a). Four was recognized as a soft-thresholding power value to create unsigned co-expression network (Fig. 1b, c), and the network contains 22 independent modules (Fig. 1d, f), which were split into two clusters (Fig. 1e). The blue module contains 869 genes, while the dark red module contains 56 genes (Additional file 1: Table S2). These two modules were identified as key modules (Fig. 2a-c). 74 hub genes and five hub genes were found in the blue and dark red module respectively (Additional file 1: Fig. S2).

### Validation of 79 hub genes

**Fig. 3:**
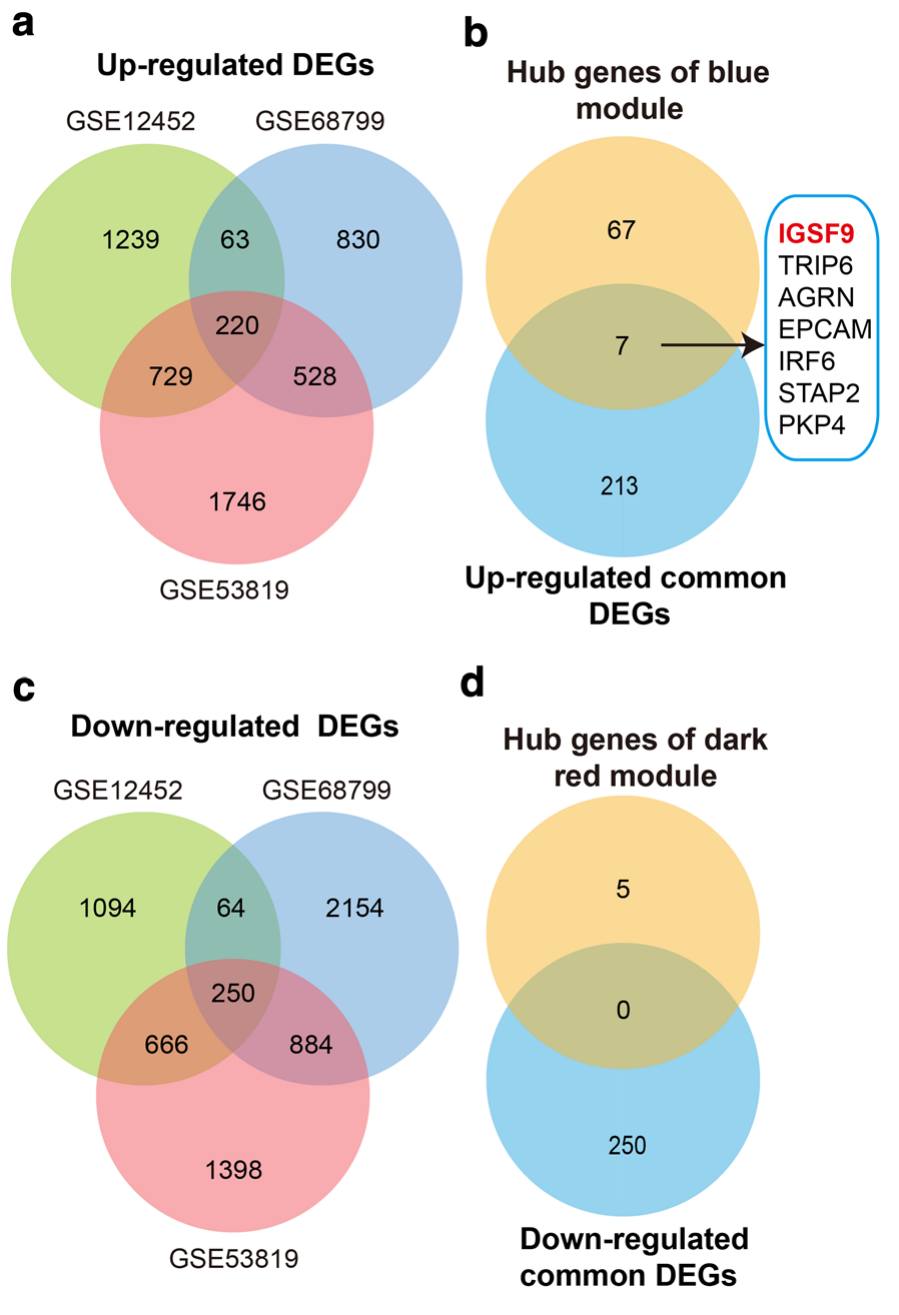
Validation of 79 hub genes. **a** Venn diagram analysis among up-regulated DEGs from three NPC data sets (GSE68799, GSE12452 and GSE53819). **b** Intersection genes of hub genes of the blue module and up-regulated common DEGs. **c** Venn diagram analysis among down-regulated DEGs from three data sets. **d** Intersection genes of hub genes of the dark red module and down-regulated common DEGs

To verify the 79 hub genes, we screened DEGs between NPC samples and non-NPC samples of three data sets (GSE68799, GSE12452 and GSE53819). 220 up-regulated common DEGs (Fig. 3a) and 250 down- regulated common DEGs (Fig. 3c) were found. Seven of 74 hub genes of the blue module were significantly up-regulated (Fig. 3b) and (Additional file 1: Fig. S3a) in the NPC patients of three data sets, while none of hub genes of the dark red module was significantly down-regulated (Fig. 3d). In addition, expression of four genes (*IGSF9*, *AGRN, STAP2* and *PKP4*) was significantly up-regulated in advanced NPC patients of the data set GSE102349 (Additional file 1: Fig. S3b), but expression of other three genes (*TRIP6, EPCAM* and *IRF6*) was not.

### Identification and functional analysis of a prognostic hub gene (*IGSF9)*

**Fig. 4:**
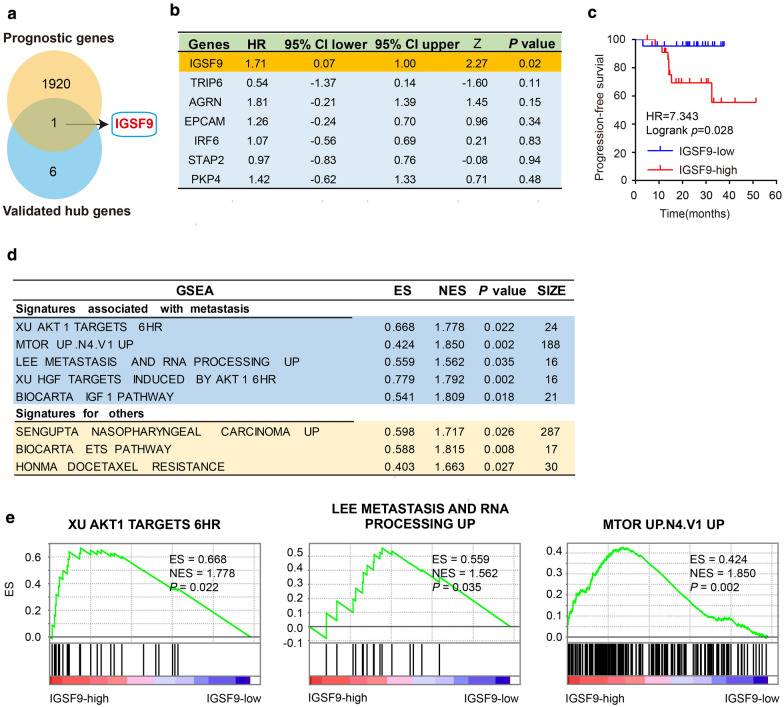
Survival and functional analysis of *IGSF9*. **a** Venn diagram analysis between validated hub genes of the blue module and genes related with PFS from GSE102349. **b** Univariate Cox regression analysis result of seven validated hub genes. Only *IGSF9* was identified as a prognostic hub gene. **c** Kaplan–Meier survival plots of *IGSF9*, which is significantly associated with the PFS for NPC patients. Patients were grouped by the trichotomization (Q1 vs Q4) value of seven genes*.* **d** GSEA results of *IGSF9* using the data set GSE102349. **e** Three significant metastatic pathways involved *IGSF9*. Absolute value of NES > 1, *P* < 0.05 were considered as statistical significance

Univariate Cox regression analysis was carried out in the data set GSE102349 using survival package in R. *IGSF9* included in seven validated hub genes was identified as a prognostic hub gene (Fig. 4a, b), and an up regulation of *IGSF9* could lead to poor PFS (Fig. 4c). Moreover, functional analysis results showed that *IGSF9* may promote metastasis of NPC cells through Akt signaling pathway, including XU AKT1 TARGETS 6HR, LEE METASTASIS AND RNA PROCESSING UP and MTOR UP. N4.V1 UP (Fig. 4d, e).

### Correlation of the scores of hypoxia signatures and the expression of *IGSF9*

**Fig. 5:**
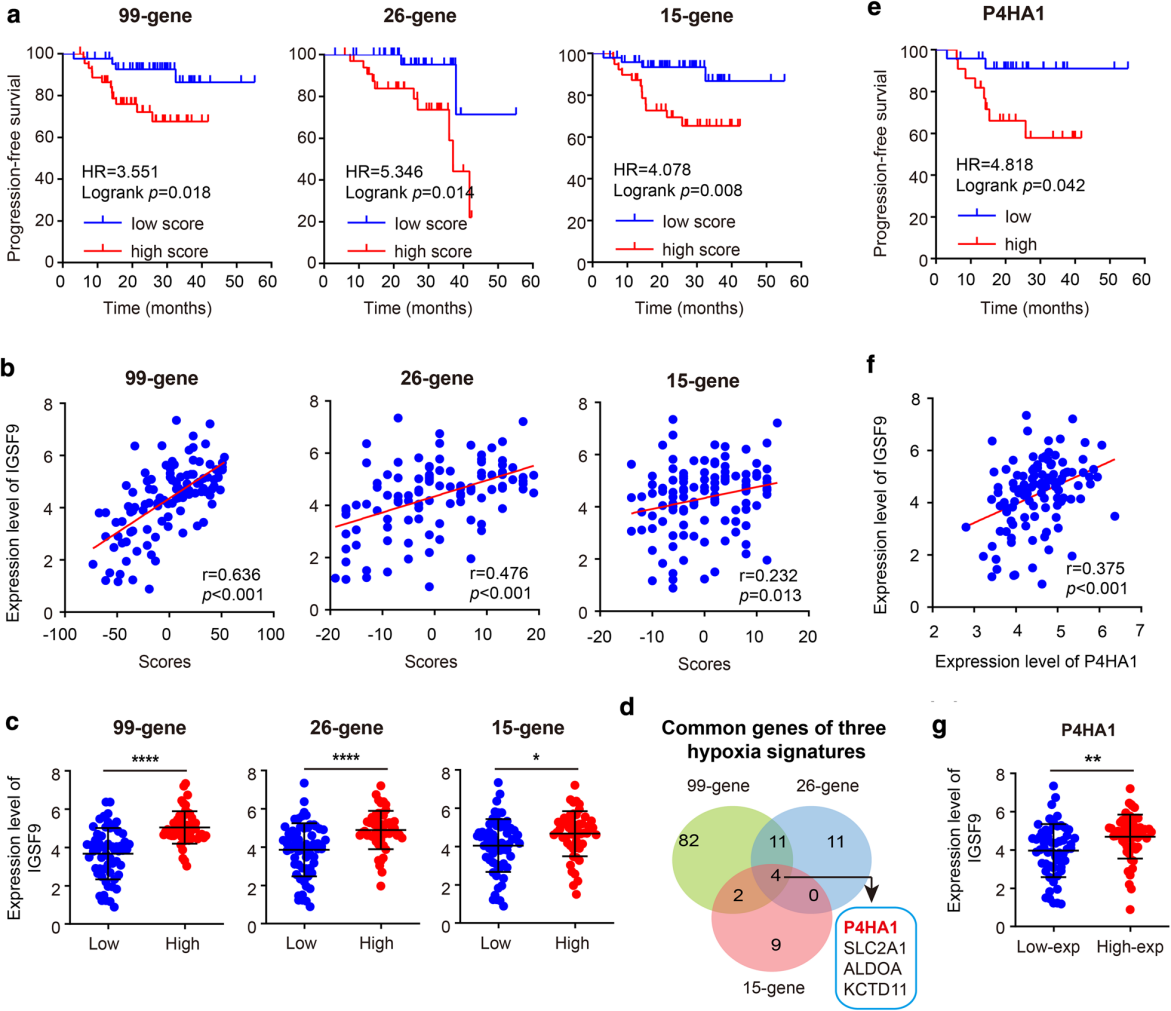
Correlation of hypoxia signatures and the expression of *IGSF9*. **a** Kaplan–Meier survival plots of three hypoxia signatures, these three signatures were related with prognosis in NPC. **b** Correlation of the scores of three hypoxia signatures and the expression of *IGSF9*. **c** Comparison results of the expression level of *IGSF9* between high and low groups according to the scores of three hypoxia signatures from the data set GSE102349. Patients used in above analysis (**a–c**) grouped by the median score of those signatures. **d** Venn diagram of genes of three hypoxia signatures.**e** Kaplan–Meier survival plots of *P4HA1*, which is significantly associated with PFS in NPC. Patients were grouped by the trichotomization (Q1 vs Q4) of *P4HA1*. **f** Correlation between  the expression level of *P4HA1*and *IGSF9*. **g** Comparison of the expression level of *IGSF9* between low-exp and high-exp group of *P4HA1* of the data set GSE102349. Patients used in above analysis (**f**, **g**) were grouped by the median value of *P4HA1*. *P < 0.05, **P < 0.01, ****P < 0.0001

The scores of three hypoxia signatures (99-gene, 26-gene and 15-gene) were calculated for survival and correlation analysis in the data set GSE102349. Patients with high scores of these hypoxia signatures had unfavorable PFS (Fig. 5a), and a parallel result was found in *P4HA1* included in four common genes (Fig. 5d, e); in other words, an over expression of *P4HA1* was associated with a poor PFS. Moreover, patients with high scores of these hypoxia signatures owned an over expression of *IGSF9* (Fig. 5b, c), which could also be seen in patients with high expression of *P4HA1* (Fig. 5f, g).

### In vitro functional analysis of *IGSF9*

**Fig. 6:**
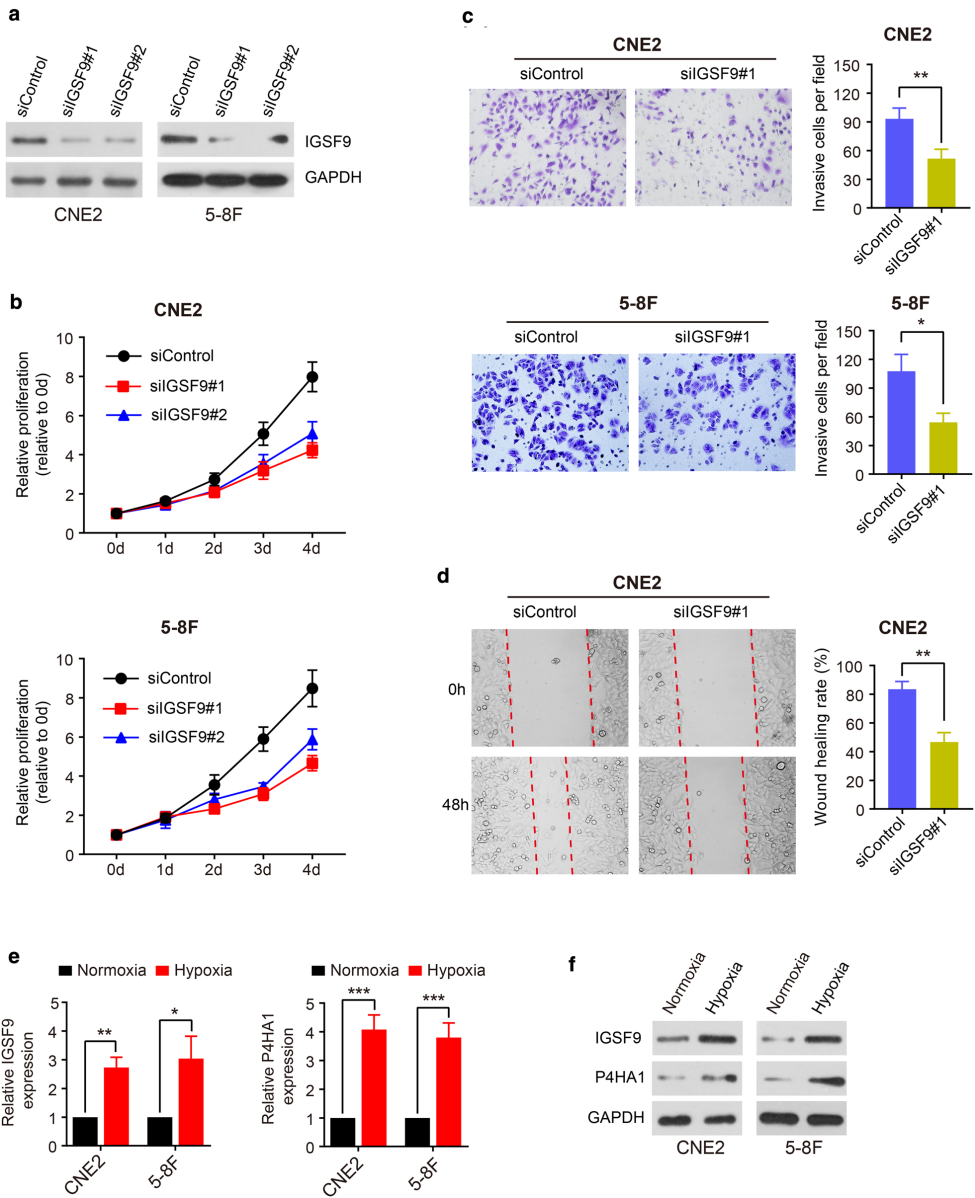
In vitro functional analysis of *IGSF9*. **a** CNE2 and 5-8F cells were transfected with siIGSF9#1 and siIGSF9#2 and subjected to western blotting for *IGSF9*. **b** Proliferative potential of two cell lines with interference of two siRNAs were analyzed by CCK8 assay. **c, d** NPC cells were treated with siRNA (siIGSF9#1) and subjected to transwell assay (**c**) and wound healing assay (**d**). **e, f **qRT-PCR (**e**) and western blotting (**f**) in CNE2 and 5-8F cells cultured under normoxia or hypoxia. All data are displayed as mean ± SD, *P < 0.05, **P < 0.01, ***P < 0.001

To investigate the effect of *IGSF9* on the aggressive phenotype, we transfected two different *IGSF9* siRNAs (siIGSF9#1 and siIGSF9#2) into CNE-2 and 5-8F cells to down-regulate *IGSF9* (Fig. 6a) and performed CCK8, wound-healing and invasion assays to evaluate whether regulation of *IGSF9* expression affects the proliferation, invasion and migration of NPC cells. The results showed a significant inhibition of proliferation, invasion and migration in the cells transfected with *IGSF9* siRNAs compared with the control cells (Fig. 6b–d). These data indicated that IGSF9 could promote NPC cell malignant biological behaviors that might be associated with poor prognosis. Next, we tried to verify the interaction between the expression of *IGSF9* and hypoxia. qRT-PCR and Western blotting were used to measure the expression of *IGSF9* and *P4HA1* in CNE-2 and 5-8F cells exposed to hypoxia. We observed that expression levels of *IGSF9* and *P4HA1* were significantly increased in NPC cells exposed to anoxic conditions (Fig. 6e, f). There was an evidence that expression of *IGSF9* might be induced by hypoxia, which was consistent with the result from our bioinformatics analysis.

## Discussion

To identify prognostic hub genes of high throughput data sets, WGCNA and univariate Cox regression analysis are very powerful tools. There are two other studies [15, 16] aiming at identifying candidate genes by WGCNA. Besides this way, our research added univariate Cox regression analysis via the mRNA-seq data set GSE102349 that contains follow-up information. By these two tools, *IGSF9* was identified as a prognostic hub gene in NPC.

*IGSF9* was highly expressed in NPC patients, and it was an appropriate prognostic gene. *IGSF9* belongs to the immunoglobulin superfamily [25], which plays a key role in inhibiting synaptic development by regulating calmodulin-like activity [26]. Calmodulin is involved in tumor metastasis, and our GSEA result also shows that *IGSF9* may cause metastasis of NPC cells through Akt signaling pathway. Meanwhile, we verified that *IGSF9* promoted the proliferation, migration and invasion of NPC cells in vitro. To date, there is one published report [27] on over-expression of *IGSF9* associated with poor PFS in endometrial cancer tissue. Similar result was found in NPC tissue in our research. Thus, *IGSF9* is likely to be a prognostic gene which would promote the invasion and metastasis of NPC cells.

Our study revealed that three hypoxia signatures (99-gene, 26-gene and 15-gene) have prognostic value in NPC patients using the data set GSE102349, though previous evidences have proved that those three hypoxia signatures can successfully discriminate the prognosis of patients with head and neck cancer [21–23, 28]. Interestingly, one research suggested that those three hypoxia signatures might be replaced by *P4HA1* [28] and another study also confirmed that *P4HA1* was a significantly prognostic factor for oral squamous cell carcinoma patients [29]. We also got a similar result in NPC patients. It is clear that our research provides valuable support for consideration of three hypoxia signatures and one single-gene (*P4HA1*) hypoxia signature as new prognostic tools like *IGSF9* in NPC patients. Moreover, we found the expression of *IGSFP* was positively associated with both the scores of three hypoxia signatures and the expression of *P4HA1* utilizing data set GSE102349, and the expression of *IGSFP* was significantly increased in NPC cells exposed to anoxic microenvironment. These data revealed that the expression of *IGSF9* in NPC cells might be affected by hypoxia.

Taken together, we identified *IGSF9* as a prognostic hub gene by WGCNA and univariate Cox regression analysis and found the expression of *IGSF9* might be induced by hypoxia. We then performed cytology experiments and verified the role of *IGSF9* in NPC cells. However, the investigation of deeper mechanism and in vivo experiment of *IGSF9* needs to be supplemented further.

## Conclusion

Our study suggests that the elevated expression of *IGSF9* will be result in a poor PFS and involved in hypoxia in NPC, implying that *IGSF9* might be expected to be a new prognostic indicator of NPC in the future.

## Supplementary information

**Additional file 1: Fig. S1.** Data distributions with or without normalization of the NPC data sets. **Fig. S2** Hub genes of two key modules. **Fig. S3** Comparison of expression of the seven validated hub genes. **Table S1** Details for the NPC data sets from GEO. **Table S2** Number of genes of 22 modules.

## Data Availability

Four data sets (GSE68799, GSE12452, GSE53819 and GSE102349) used in this article were derived from in GEO database.

## Abbreviations

NPC: Nasopharyngeal carcinoma
LDH: Lactate dehydrogenase
WGCNA: Weighted gene co-expression network analysis
GEO: Gene expression omnibus
VAR: Variance analysis report
TOM: Topological overlap measure
MEs: Module eigengenes
MM: Module membership
GS: Gene significance
DEGs: Differentially expressed genes
HR: Hazard ratio
PFS: Progression-free survival
GSEA: Gene set enrichment analysis
NES: Normalized enrichment score
FBS: Fetal bovine serum
qRT-PCR: Quantitative real time polymerase chain reaction
CCK8: Cell counting kit 8
SD: Standard deviation
