# Supplementary material for: Identified IGSF9 association with prognosis and hypoxia in nasopharyngeal carcinoma by bioinformatics analysis
Source: Cancer Cell Int. 2020 Oct 12;20:498. doi: 10.1186/s12935-020-01587-z (PMC7552377; doi:10.1186/s12935-020-01587-z)
Supplement: Supplementary file 1 — Additional file 1: Fig. S1. Data distributions with or without normalization of the NPC data sets. Fig. S2 Hub genes of two key modules. Fig. S3 Comparison of expression of the seven validated hub genes. Table S1 Details for the NPC data sets from GEO. Table S2 Number of genes of 22 modules. [file 12935_2020_1587_MOESM1_ESM.pdf]

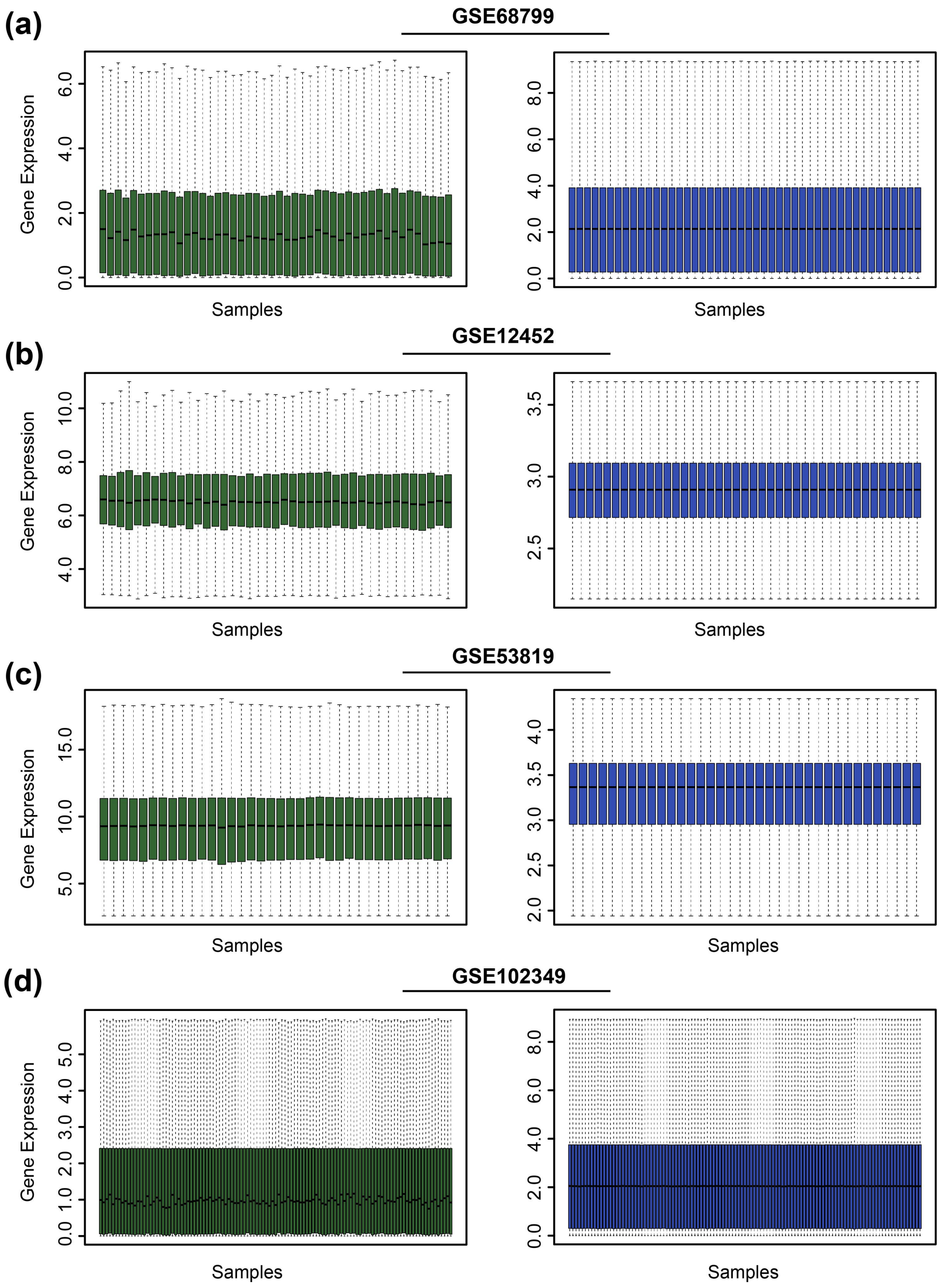

Fig. S1 Data distributions with or without normalization of NPC data sets. (a-d) Normalization of data set GSE68799 **(a)**, GSE12452 **(b)**, GSE53819 **(c)**, GSE102349 **(d)**. Green box-plot shows the data distributions without normalization, and blue box-plot shows the data distributions with normalization.

(a) **Hub gene of blue module**

(d) **Hub gene of darkred module**

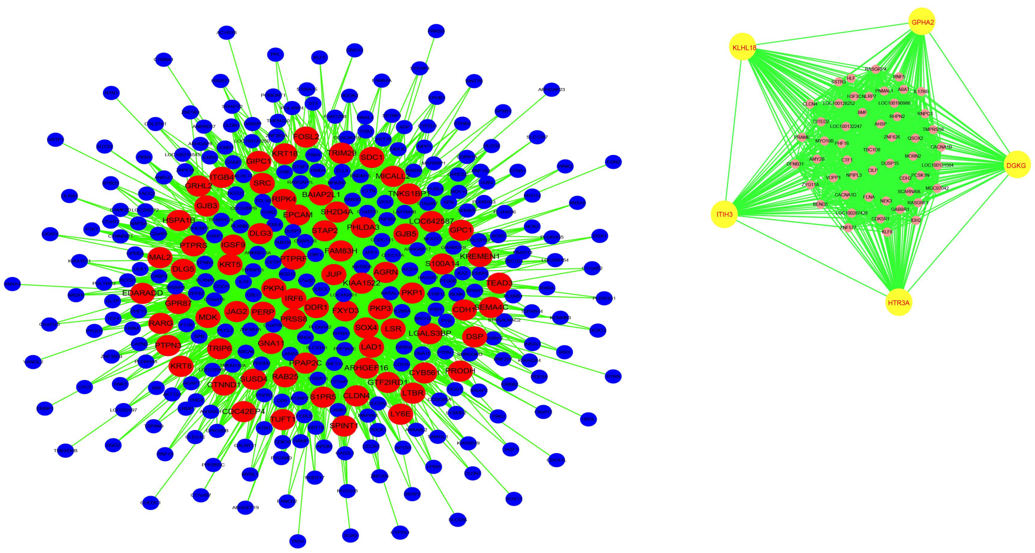

Fig. S2 Hub genes of two key modules. (a) Hub genes (74 red nodes) of the blue module. **(b)** Hub genes (five yellow nodes) of the dark red module.

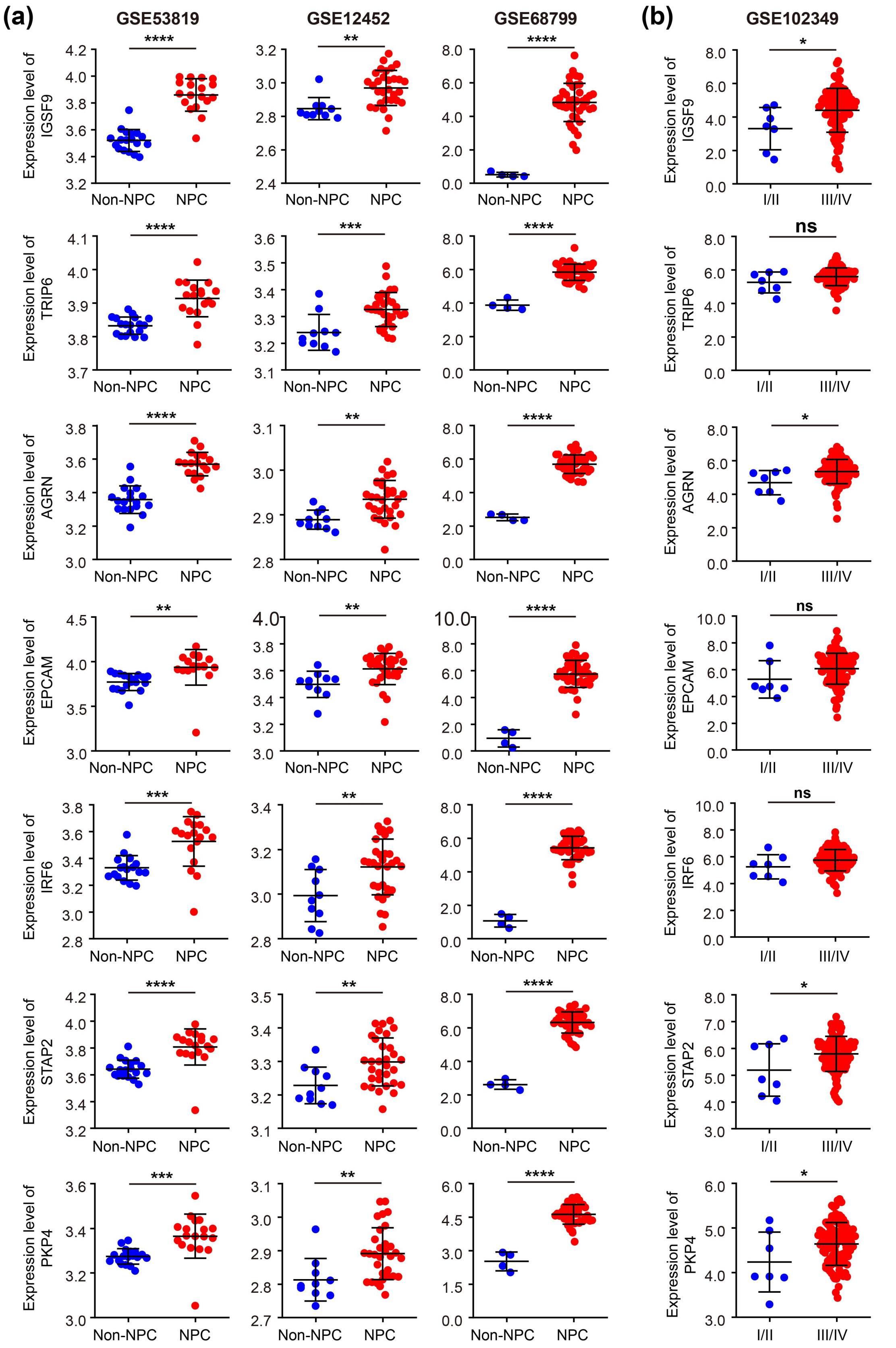

Fig. S3 Comparison of expression of seven validated hub genes. (a-b) Expression of seven genes (*IGSF9, TRIP6, AGRN, EPCAM, IRF6, STAP*2 and *PKP4*) between NPC and non-NPC samples in three data sets (GSE68799, GSE12452 and GSE53819) **(a)** and between different clinical stages in data set GSE102349 **(b)**. *$P < 0.05$, **$P < 0.01$, ***$P < 0.001$ and ****$P < 0.0001$.

Table S1 Details for NPC data sets from GEO

Series ID	Platform	NPC	Non-NPC	Country
GSE68799	GPL11154	42	4	China
GSE12452	GPL570	31	10	USA
GSE53819	GPL6480	18	18	China
SE102349	GPL11154	113	0	USA

Table S2 Number of genes of 22 modules

Module name	Number of genes	Module name	Number of genes
magenta	255	green yellow	171
turquoise	869	red	320
purple	192	light green	90
brown	551	light cyan	111
cyan	119	grey60	93
blue	639	royal blue	55
green	415	salmon	154
midnight blue	114	dark green	53
pink	255	light yellow	80
yellow	441	tan	163
black	263	dark red	56
